# Hirschsprung Disease: The Role of the Clinical Nurse Specialist

**DOI:** 10.3390/children11050587

**Published:** 2024-05-13

**Authors:** Julie-Ann Milbery, Joe Curry

**Affiliations:** Great Ormond Street Hospital for Children NHS Foundation Trust, London WC1N 3JH, UK; joe.curry@gosh.nhs.uk

**Keywords:** Hirschsprung disease, clinical nurse specialist, support, continence

## Abstract

Hirschsprung disease is a life-long condition that can have a significant impact on both children and their families. This article explores the role of the clinical nurse specialist and the support they can provide from initial diagnosis through the patient’s surgical journey and right through to transition into adult services. Through the provision of education, training, signposting of social and psychological support, and linking in with community-based services, the clinical nurse specialist can help the child and family to limit that impact of the disease.

## 1. Introduction

**Figure 1:**
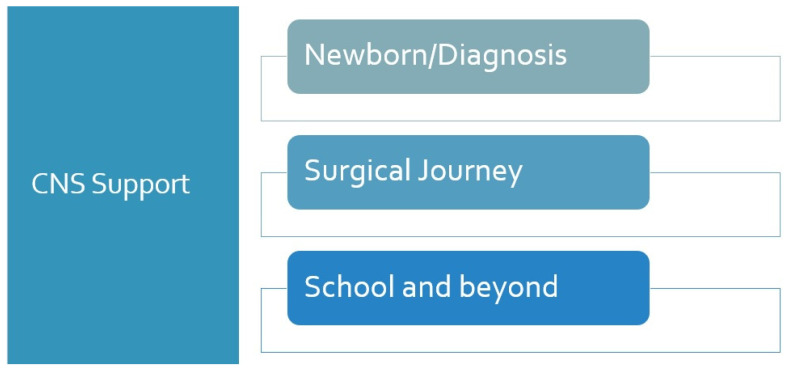
Involvement of specialist nurse team in journey of Hirschsprung.

Even though Hirschsprung disease (HD) is a single-organ disease, its impact on the child and family is varied and widespread. It is known that conditions affecting continence can have a significant impact on childhood quality of life and development [1,2]. Management of the condition is best undertaken in the context of a wide multidisciplinary team. Increasingly recognised within the experienced multidisciplinary team caring for children with HD is the presence and function of the clinical nurse specialist [3]. They can provide a vital interface between the family and their needs and requirements while outside of hospital and the in-hospital medical team. They are vital in the support of the family as they deal with the trauma of understanding the new diagnosis and its implications for their child [2,4]. They can support the family through the early phase of the surgical journey, and importantly, they are there to help and support the family in their journey beyond, right through to their point of transition (Figure 1). Within the short article, we will define some of the key roles of the clinical nurse specialist and underpin the necessity of their presence in any multidisciplinary team treating children with this condition. Currently within the UK, there is no recognised education program/qualification required for a registered nurse to develop her role or apply for a post as a clinical nurse specialist; however, a number of years’ experience in the necessary field is essential.

## 2. Diagnosis

Parents are immediately dealing with the trauma of understanding that their baby may have a serious condition at birth or in the ensuing months. The clinical nurse specialist (CNS) has the role of supporting and reassuring the family. In very practical terms, this can be in supporting the parents’ learning of how to undertake the process of rectal washout to support adequate decompression and in facilitating their ability to feed the child orally [5]. Once the diagnosis is established, a pathway can then be set for the management of the child, which is usually well known to the medical team and can be related back to the family. This, of course, can bring many challenges, particularly, for example, if there is only one parent in the family or if there are other barriers such as problems with language, limited family support or income, and parents who themselves may have some learning difficulties and/or difficulties with their own mental health.

It may be necessary to tailor individual packages of care for families as their babies move towards discharge from the initial admission. In the context of tailored packages for families with some difficulty, the involvement of family social worker within the hospital can be vital, and this is again facilitated by the multidisciplinary team (MDT) support provided by the CNS. There may be charitable funds available within the context of the organisation to support families both in and out of hospital. The family social worker will be able to provide potential support for subsistence while in the hospital, as well as travel costs to and from home where necessary. Within the UK, it is possible to apply for disability living allowance, which can provide additional financial support as well as practical support, such as access to disabled toilets, for example [6].

## 3. Discharge Home

Once the adequacy of washouts has been confirmed and the parents have been suitably trained and assessed, discharge can be achieved. The CNS will discuss with the family the red flags of concern that would necessitate immediate return for review and provide the family with an array of contact details (both telephone and email) to ensure that family has as a safety net. The CNS will also inform the local community teams and family practitioner that a child with a new diagnosis of Hirschsprung disease has been released into their joint care in the community and, similarly, provide them with red flags of concern. The child will be returned to the ward on a 1–2 weekly basis to review the continued effectiveness of washouts and ensure that the child is thriving and putting on weight. If the child has required a stoma, then, again, the adequacy of parental care of the stoma will be established prior to discharge. The child will be handed over to the community team, who will continue to provide support and access to appliances. It will be necessary to liaise with local paediatric medical support so they are aware of the patient and their diagnosis in case there is a requirement for an emergency readmission.

## 4. Surgery

**Figure 2:**
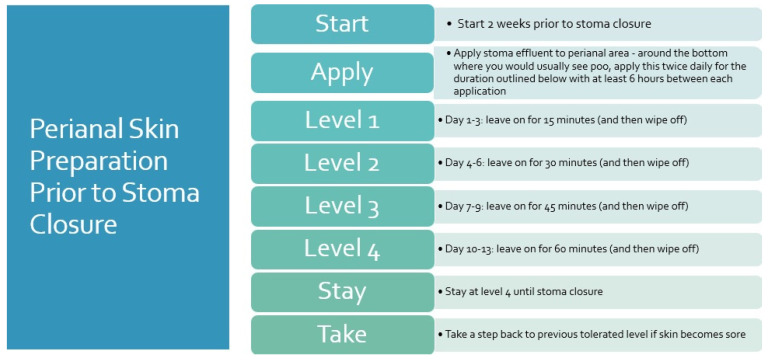
Perianal skin preparation prior to stoma closure.

The time leading up to surgery can be a period of stress and concern for families, and many will seek reassurance and further guidance from the CNS at this time. Information provided from the medical team can be complex and overwhelming, and the CNS’s confirmation and reinforcement of this information over time is valuable in adding to the parents understanding [7]. If the child is having reconstruction following the institution of a stoma, then the resumption of faecal effluent onto the perineum can result in significant excoriation. We have developed a protocol for perineal skin management and protection, which is discussed with the family, and appropriate skin protection is commenced in the immediate post-operative period. At our institution, we have been advising parents to apply stoma effluent to the perineum on an increasing time basis to prepare the skin in an effort to prevent post-stoma-closure perianal dermatitis when the skin will initially be regularly exposed to effluent. The CNS has facilitated this process, showing some early signs of improvement in post-operative perianal excoriation (Figure 2).

## 5. Bowel Management

Children with HD will, at some stage in their journey, have a reconstructive procedure as part of their management. The specific details of the reconstruction are beyond the scope of this text. One of the most important roles of the CNS is in initiating and supporting effective bowel management for children following reconstruction. An effective bowel management plan has to be based on a recognised protocol but ultimately tailored to the individual needs of the child and the environment they are in [8]. Supporting parents in delivering this and, importantly, in assessing the outcome of this is an important role within the MDT. It is important for the family to have realistic expectations set and then for them to be able to relay their plans and outcomes to the MDT via the CNS. Ultimately, this is a role in which the CNS can work autonomously with the family to achieve set and agreed aims. Diaries of stool frequency and type (using a model such as the Bristol stool chart) are vital in assessing the ongoing effectiveness of the bowel management programme [9].

## 6. Potty Training

As the time of potty training approaches, many parents are understandably anxious [10]. It may not, at this stage, be fully possible to understand the child’s potential ability to be normally and naturally continent. This is when families will require the help and support of the CNS to work through this period. Setting appropriate expectations is vital as clearly, for some children, potty training may be impossible. The CNS can support families through establishing simple routines for potty training and utilising natural physiological phenomena such as the gastrocolic reflex. It is likely to also be a time when there will be some adjustments to laxative regimes to facilitate the stimulated evacuation of faeces. If the possibility of continence is not achievable or not occurring in time, then the CNS will be vital, ensuring that the family have adequate support for continence services, and this is often a time when clinical psychological support can be valuable to support parents and children.

## 7. School

**Figure 3:**
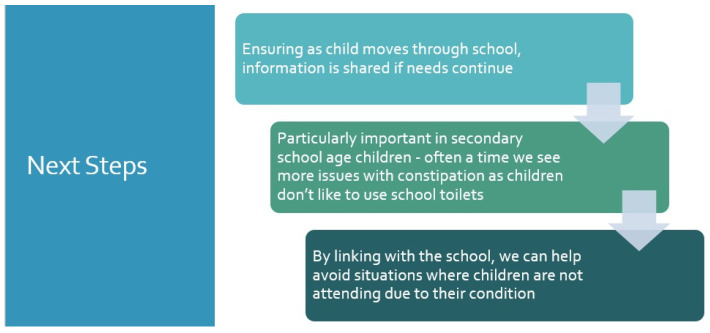
CNS support to help with school attendance.

School is another important milestone, leading to a sense of frustration and anxiety for many parents with children with continence difficulties. Ensuring that the educational environment is aware of a child’s needs from a continence perspective is important and is a key role of the CNS within the MDT. This role can be in supporting families in delivering this information to schools, delivering some form of information through written documentation of the child’s medical needs, and often meeting with the school virtually to discuss the child’s healthcare needs with the teachers and classroom assistants (Figure 3). Some very simple plans can be put in place that can transform children’s confidence within the school environment particularly in year 7 and above (age 11+). These can be in the form of agreed criteria for the child to be able to leave the classroom environment and for the child to be able to use safe and private toileting facilities such as those used by the teachers.

It is well known that children with continence issues can miss significant time away from their time in primary school, and the role of the CNS is to ensure that this time away because of continence is kept to an absolute minimum [1]. Within the UK, there is legislation to support children’s needs within the classroom environment in the form of an educational health care plan (EHCP) [11]. This legal document sets out any additional needs the child has as a result of their medical condition. If an EHCP is awarded, specific funding is provided to ensure that adequate resources are available to support the child’s additional needs in the classroom environment. It is not required for every child, but the CNS can support the family through looking at this particular process.

## 8. Beyond to Transition

The teenage years for children with continence difficulties present new challenges [12,13,14]. Children are developing a sense of their own identity and for many, for the first time, they are starting to understand the impact of their diagnosis on their quality of life, social functioning, and development of personal relationships. Parents find these challenges difficult and will frequently reach out to the clinical nurse specialist as the person they have known and trusted through their child’s development for help and support. The involvement of clinical psychology at this stage is also important for support and therapy to ensure a smooth transition. Processes of transition within the trust will be known to the CNS, who can be part of the process of identifying an individualised pathway of transition and can work closely with adult colleagues to ensure a smooth hand over.

## 9. Conclusions

Hirschsprung disease, for many, is a condition that can be effectively treated with reconstruction leading to an excellent quality of life. For others, however, it brings potential lifelong issues of continence difficulties. A clinical nurse specialist has the unique ability to share in this journey from birth to transition and provide an effective interface between the clinical in-hospital environment and the day-to-day life of home and school. To objectively measure the true impact of the CNS on the journey of both children and their families would be incredibly challenging; what has been outlined in this article is based on our experience and parental feedback. The quality and value of care that the CNS is able to provide can ensure that children continue to develop and learn as any child should and prevent them from being defined by their medical condition.
